# Enhanced CPU Design for SDN Controller

**DOI:** 10.3390/mi15080997

**Published:** 2024-07-31

**Authors:** Hiba S. Bazzi, Ramzi A. Jaber, Ahmad M. El-Hajj, Fathelalem A. Hija, Ali M. Haidar

**Affiliations:** 1 Electrical and Computer Engineering Department, Beirut Arab University, Debieh 1504, Lebanon; ari@bau.edu.lb; 2 Electrical and Electronic Engineering Department, Lebanese University, Hadath 40016, Lebanon; ramzi.jaber@ul.edu.lb; 3 Olayan School of Business, American University of Beirut, Beirut 1107, Lebanon; ae37@aub.edu.lb; 4 Cyber Security Graduate Program, Joaan Bin Jassim Academy for Defence Studies, Al Khor 50819, Qatar; fali@jbj.edu.qa

**Keywords:** CNTFET, ternary logic design, software-defined networking (SDN), unary operators, programmability

## Abstract

Software-Defined Networking (SDN) revolutionizes network management by decoupling control plane functionality from data plane devices, enabling the centralized control and programmability of network behavior. This paper uses the ternary system to improve the Central Processing Unit (CPU) inside the SDN controller to enhance network management. The Multiple-Valued Logic (MVL) circuit shows remarkable improvement compared to the binary circuit regarding the chip area, propagation delay, and energy consumption. Moreover, the Carbon Nanotube Field-Effect Transistor (CNTFET) shows improvement compared to other transistor technologies regarding energy efficiency and circuit speed. To the best of our knowledge, this is the first time that a ternary design has been applied inside the CPU of an SDN controller. Earlier studies focused on Ternary Content-Addressable Memory (TCAM) in SDN. This paper proposes a new 1-trit Ternary Full Adder (TFA) to decrease the propagation delay and the Power–Delay Product (PDP). The proposed design is compared to the latest 17 designs, including 15 designs that are 1-trit TFA CNTFET-based, 2-bit binary FA FinFET-based, and 2-bit binary FA CMOS-based, using the HSPICE simulator, to optimize the CPU utilization in SDN environments, thereby enhancing programmability. The results show the success of the proposed design in reducing the propagation delays by over 99% compared to the 2-bit binary FA CMOS-based design, over 78% compared to the 2-bit binary FA FinFET-based design, over 91% compared to the worst-case TFA, and over 49% compared to the best-case TFAs.

## 1. Introduction

SDN revolutionizes network management by separating the control plane from the data plane. In SDN, software-based controllers manage network traffic efficiently, interacting directly with the physical hardware. This approach is gaining global traction, with significant investments in research and development. SDN represents the future of networking, transforming traditional concepts into more flexible, compatible, and easily maintainable systems [1,2].

Consolidating network control is the core innovation of SDN. All network devices are managed by a centralized controller rather than by separate devices acting independently. This method reduces the overall complexity of the network and enhances scalability. Furthermore, this centralization promotes devices from various vendors to function seamlessly together, going beyond network administration and infrastructure

Novel technologies like software-defined storage and data center administration from a single point of control were made possible by SDN [3]. Security management becomes more effective with the centralized monitoring and control provided by SDN. Furthermore, SDN makes Network Function Virtualization (NFV) easier by centralizing load balancing, firewalls, and DNS, which lowers the necessity for a lot of hardware [4,5].

**Figure 1:**
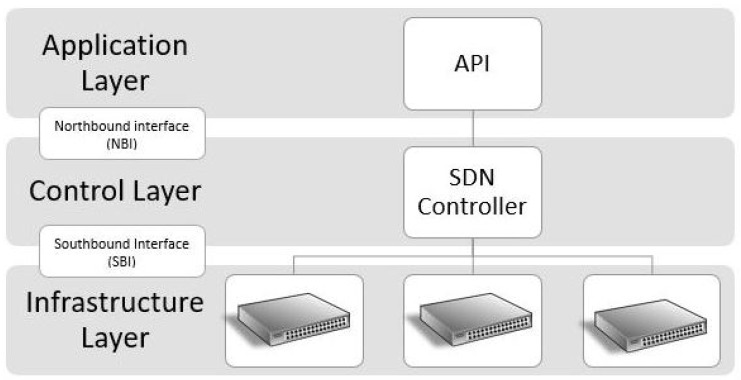
SDN architecture.

The OpenFlow protocol facilitates communication between the controller and network devices, serving as both a protocol and architecture for SDN. The architecture includes a controller and OpenvSwitch, which uses OpenFlow and other southbound protocols as shown in Figure 1. OpenvSwitch features components like secure communication channels, flow tables for packet forwarding, and meter tables for quality of service. OpenFlow’s open standard allows interoperability among different vendors’ controllers and switches.

Overall, SDN’s centralization and standardization, exemplified by protocols like OpenFlow, represent a significant leap forward in networking, enhancing flexibility, scalability, and security. However, several key points must be considered before choosing the hardware for the SDN controller, depending on the specific demands of the SDN deployment [6,7]. Some essential factors to consider are the CPU, Memory (RAM), Networking Interfaces, Redundancy and high availability, and scalability [8]. Among these, the CPU plays a pivotal role in the operation of the controller in SDN. Therefore, to meet the demands of network management, the CPU must be a high-performance processor, capable of managing network traffic and policy enforcement.

The problem of CPU utilization is identified as a significant challenge in SDN environments [9,10]. The increased inflow of packets requiring processing in the control plane puts strain on the CPU, affecting overall network performance. To address this problem, the paper proposes a ternary system to improve the CPU inside the controller to enhance network management.

The multiple-valued logic (MVL) circuit shows remarkable improvement compared to the binary circuit regarding the chip area, propagation delay, and energy consumption [11,12,13]. Specifically, the ternary system has the best efficiency regarding the circuit complexity and cost compared to other bases, as proved mathematically by the authors of [14,15,16].

There are two methods to express ternary logic systems. The first one is unbalanced: (0, 1, 2) corresponds to (0, Vdd/2, Vdd). The second one is balanced: (−1, 0, 1) corresponds to (−Vdd, 0, Vdd).

For example, the eight-digit decimal number (34567890) is (10 0000 1111 0111 0110 1101 0010) 26 bits in binary, whereas the ternary equivalent is 16 trits (2102 0010 2002 0020). Thus, if the reduction in wiring for just 26 bits is around 38.46%, imagine the reduction percentage regarding much larger numbers of bits.

Almost 90% of digital circuits use CMOS transistors because they are cheaper than other transistor technologies. The CNTFET has provided the best trade-off regarding the circuit speed and energy efficiency compared to different transistor technologies [17,18]. More details about the CNTFET are found in the Materials and Methods section below.

### 1.1. CPU Utilization and Importance of Programmability in SDN

Programmability is a crucial aspect of SDN, allowing for dynamic and adaptive network operations. This separation enables centralized control and the implementation of complex traffic management, routing, and security algorithms without altering the underlying hardware. SDN programmability is achieved through combining the interface infrastructure, controller, and API layers [19,20]. Applications can use APIs to programmatically create and modify network policies, thus dynamically controlling network behavior. The controller translates these policies into commands at the infrastructure layer, enabling quick service deployment, efficient resource allocation across the network, and the flexibility to adapt to changing demands. This flexible and programmable framework helps network administrators gain the ability to automate and improve various network management tasks.

Moreover, the programmability of SDN significantly impacts CPU usage. With centralized and dynamic SDN control, CPU resources are used more efficiently for processing and enforcing network policies, managing traffic, and executing security protocols. Centralizing these operations reduces CPU usage and enhances overall network efficiency.

### 1.2. Factors Contributing to High CPU Utilization in SDN

In SDN, several factors can contribute to high CPU utilization. The following are some of the common causes:

**Control Plane Processing:** The control plane manages network policies, packet forwarding decisions, and network events [20]. CPU usage can spike during complex calculations or high volumes of control messages, especially in large networks or during frequent network changes.

**Flow Table Updates:** Flow tables in SDN switches dictate packet processing and forwarding. Frequent updates due to network events or policy changes increase the CPU load as the controller processes and communicates these updates to the data plane [21].

**Packet-in Events:**When a packet lacks a matching flow table entry, it is sent to the controller for handling. Managing many packet-in events can significantly raise CPU utilization [22].

**Network Monitoring and Analytics:** Real-time monitoring, traffic analysis, and enforcing security policies in SDN require processing extensive network data and running complex algorithms, consuming substantial CPU resources [22].

**Controller Scalability:** In large SDN deployments, the controller must manage numerous control messages, flow table entries, and communications with many devices, increasing CPU utilization as network complexity grows [23].

### 1.3. Programmability vs. CPU Utilization

Table 1 outlines various aspects affecting the programmability and CPU utilization in SDN where Control logic flexibility allows easy updates with moderate CPU usage based on task complexity. High programmability enables frequent policy updates, leading to high CPU usage, and supports complex algorithms, increasing CPU usage due to intensive computations. Dynamic flow management results in moderate to high CPU usage due to frequent updates. High programmability in network analytics enables detailed monitoring and reporting, consuming significant CPU resources. Additionally, flexible device interfacing results in moderate CPU usage. This table shows the necessity for a powerful CPU in an SDN controller to handle the diverse and demanding tasks brought by programmability [24]. Enhanced CPU designs, especially those incorporating advanced techniques like ternary logic in the ALU, can significantly improve the efficiency and performance of SDN controllers, enabling them to better meet the dynamic needs of modern networks. By emphasizing programmability and optimizing CPU utilization, particularly through innovations in ALU design, we can enhance the capabilities of SDN controllers to manage complex network environments more effectively [25].

The rest of the paper is organized: The Literature Review is presented in Section 2. Materials and methods in addition to the background of some ternary circuits and CNTFETs are presented in Section 3. Section 4 describes the proposed new TFA. Section 5 discusses the simulation results and comparisons. Finally, the conclusion is in Section 6.

## 2. Literature Review

Several studies have examined the impact of CPU utilization in the context of SDN by conducting a comprehensive analysis of CPU usage in SDN controllers, highlighting the factors contributing to high CPU utilization.

The authors of [26] discussed the issue of CPU utilization in the context of an integrated architecture of SDN and Software-Defined Radios (SDRs) for cloud-based communication systems. The authors analyze the CPU utilization and power consumption in the OpenIreland testbed and compare the behavior of SDN data plane switching and SDRs. They propose a power-saving scheme with flexible CPU allocation to reduce overall power consumption. The experimental results show that the proposed architecture and power-saving scheme can save up to 20% of power consumption compared to the conventional approach where SDN and SDRs are separately deployed. The paper highlights the different CPU utilization features of SDN and SDRs, emphasizing the potential for power savings through an integrated CPU deployment. However, specific percentages of improvement are not mentioned in the brief document provided.

Moreover, the authors of [27] presented energy-efficient techniques in SDN and classified them into software-based, hardware-based, and hybrid approaches. The authors highlight the challenge of optimizing energy consumption while maintaining network performance. One of the specific areas of concern is CPU utilization within SDN. The paper explored a range of techniques aiming to tackle this issue. These techniques, including traffic awareness, end-host awareness, and rule placement, are thoroughly examined by the authors. Their primary objective is to effectively manage CPU usage in SDN networks by dynamically controlling traffic flow and routing policies. The authors emphasize the importance of optimizing CPU utilization and programmability to achieve energy efficiency in SDN.

Furthermore, the authors of [28] addressed the problem of layer 2 loop prevention in software-defined networks (SDNs) and proposed a new method that utilizes the global view of the SDN controller. The traditional Spanning Tree Protocol (STP) used in legacy networks is inefficient for larger networks and lacks programmability. The proposed method aims to reduce CPU utilization on the controller and improve network performance by blocking fewer switch ports, utilizing more capacity for switches, and decreasing link recovery time. By leveraging the centralized control plane of SDN, the method prevents broadcast storms and loop formation, providing a more efficient and scalable solution compared to STP.

On the other hand, numerous articles suggested various methods to design TFAs based on CNFETs to enhance CPU performance. Table 2 provides an overview of the various methodologies proposed in recent articles with their respective limitations.

The authors of [29] use 2-bit binary Full Adder and implement the circuit with two-transistor technology: 250 CMOS and 250 FinFET. Binary circuits will generate high propagation delays and PDP.

The conventional design in the ternary system can be implemented by transforming ternary inputs into intermediate binary bits through Ternary Decoders (TDecoders), and, subsequently, utilizing binary gates followed by ternary encoders to generate the targeted ternary outputs. This approach will generate a high transistor count and PDP, as indicated in the referenced papers:

In [30], the authors proposed a TFA with 412 CNFETs and the authors of [31] showed a TFA with 337 CNFETs and 14 RRAMs (Resistive Random Access Memories).

Another approach used algorithms for logic synthesis, which will result in a high number of transistors connected in series, resulting in high propagation delays and PDPs. The papers referenced in support of this methodology are as follows:

In [32], the authors used two custom algorithms to generate a TFA consisting of 105 CNFETs. These algorithms were specifically designed to generate unary operators and enable the cascading of TMUXs in the TFA design. The authors of [33] presented a TFA implementation utilizing a Ternary-Transformed Binary Decision Diagram (TBDD) algorithm, resulting in a TFA with 98 CNFETs. In contrast, the authors in [34] demonstrated a TFA design with 106 CNFETs by employing a modified Quine–McCluskey algorithm and post-optimization algorithms.

Another technique used unary operators of the ternary system combined with TMUXs that proved to be effective in generating a low transistor count and minimizing PDPs. The following articles demonstrate the utilization of this approach:

The authors of [35] designed a Ternary Full Adder (TFA) with 74 CNFETs, while the authors of [36,37] designed TFAs with 89 and 72 CNFETs, respectively.

Finaly, the following papers employed a combination of different techniques:

In [39], the authors put forth a TFA design comprising 142 CNFETs. This design leverages ternary encoders, unary operators based on Binary NAND, and TMUXs. In [41], the authors introduce a TFA with 74 CNFETs. This design incorporates Pass Transistor Logic (PTL) and TMUXs, resulting in moderate propagation delays and a corresponding medium power-delay product (PDP). Additionally, in [42] the authors proposed a TFA design consisting of 54 CNFETs. This design employs a combination of Transmission Gates, TDecoders, unary operators, and PTL to achieve its functionality.

The literature review analyzes and explores the limitations associated with CPU utilization and programmability in SDN. The reviewed studies highlight the challenges faced in managing CPU usage during control plane processing, flow table updates, and packet-in events. To address these challenges, various mitigation strategies are proposed, including flow table caching and hardware offloading, which aim to alleviate the burden of high CPU utilization.

### Contributions

Controllers’ CPUs encounter several critical limitations. The heavy load associated with processing numerous control messages and flow entries often results in performance bottlenecks and latency issues. Moreover, scalability is another challenge, as larger networks place greater demands on the CPU’s limited processing power and memory. Additionally, CPUs within SDN systems consume significant power and produce heat, increasing the need for cooling and energy. The complexity of advanced algorithms and software overhead further burdens CPU resources.

A ternary full adder offers a solution to these limitations by reducing latency and enhancing overall performance by increasing information density and processing efficiency. Ternary logic optimizes resource and processing power usage, improving scalability and enabling CPUs to manage larger networks more effectively. Enhanced error detection and correction capabilities of ternary logic improve fault tolerance and reliability, while its complexity strengthens defenses against intrusions.

Ternary has been added to SDN in numerous research publications because of its substantial influence on overall network performance. Nevertheless, the majority of these investigations have concentrated on high-speed TCAM in SDN [43,44], ignoring the possible improvements to the CPU, specifically the ALU design. Improving an SDN controller’s CPU can analyze information and make choices more quickly. Reducing the complexity and increasing the speed of the ALU design with ternary logic is crucial for handling the real-time demands of SDN environments. This improvement not only fixes existing issues but also opens the door for SDN controllers that are more sophisticated and responsive.

This paper will focus on the enhancement of the CPU inside the SDN controller by using a ternary logic system rather than a binary logic system and also by implementing a CNTFET rather than a complementary metal-oxide-semiconductor transistor (CMOS) or fin field-effect transistor (FinFET).

Therefore, this paper proposes the TFA using CNTFETs, an unbalanced ternary logic system (0 V, 0.45 V, 0.9 V), and two supply voltages (Vdd and Vdd/2).

The new design offers a significant improvement; it has the lowest propagation delay and energy consumption compared to the designs in [29,30,31,32,33,34,35,36,37,38,39,40,41,42], as evidenced by the simulation results using HSPICE.

## 3. Materials and Methods

This paper proposes the Stanford CNTFET-based TFA using a Ternary Multiplexer (TMUX) with unary operators.

### 3.1. CNTFET Transistor

More details about the Stanford CNTFET model are found in [45,46,47]. However, it is necessary to note that the threshold voltage depends on the diameter of the carbon nanotube (Dcnt), as shown by Equation (1):

(1) $$V_{\mathrm{th}}=\frac{0.43}{Dcnt}$$

Table 3 displays the operation of the CNTFET and presents the relationship between the threshold voltage and the CNT diameter, which this paper uses in the design.

### 3.2. Unary Operators

One-input and one-output logic gates are called unary operators of *p*-valued systems [48].

For *p* = 2 (a binary system), there are four ($2^{2}$) unary functions (“00”, “01”, “10”, “11”). However, for *p* = 3 (a ternary system), there are 27 ($3^{3}$) unary functions (“000”, “001”, “002”, …, “220”, “221”, “222”).

Table 4 shows seven unary functions presented in [38,49,50] that are implemented in the design.

### 3.3. Ternary Multiplexer

**Figure 2:**
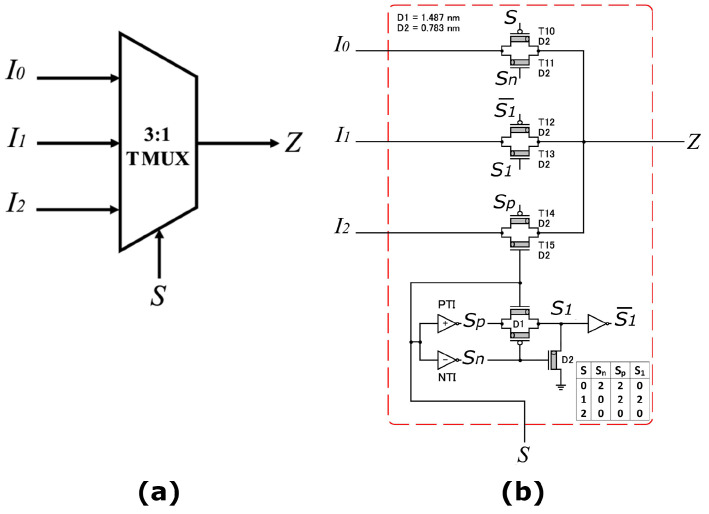
The (3:1) TMUX with 15 CNTFETs [49]: (**a**) The General Model; (**b**) The Transistor Level.

**Figure 3:**
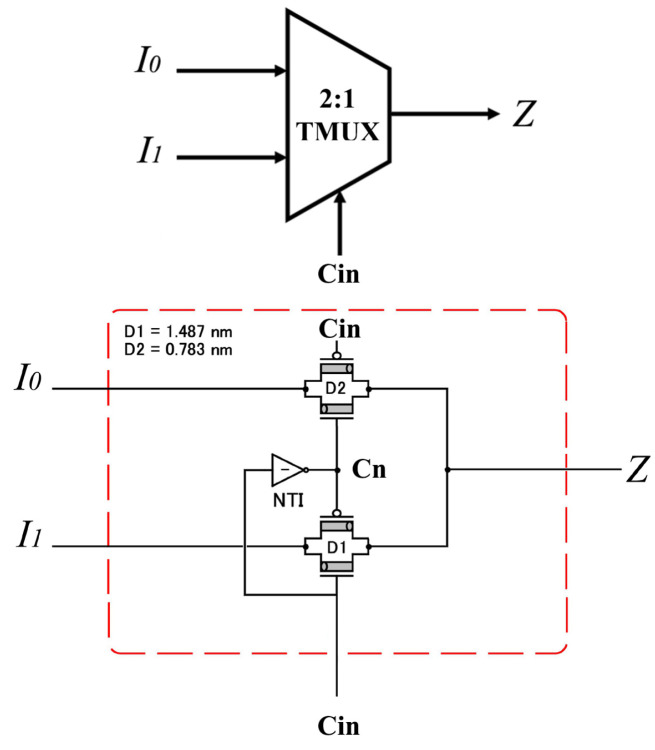
Special (2:1) TMUX for selector $C_{in}$.

Two Ternary Multiplexers (TMUXs) are used: (a) (3:1) TMUX in [49], and (b) (2:1) TMUX in [38].

(a)—The (3:1) TMUX in [49] has three inputs ($I_{0}$, $I_{1}$, $I_{2}$), one selector (*S*), and one output (*Z*), as shown in Figure 2 and described in Equations (2) and (3):

(2) $$Z=I_{0}.S_{0}+I_{1}.S_{1}+I_{2}.S_{2}$$

(3) $$Z=\left\{\begin{array}{cc} I_{2}, & if\;S=2\\ I_{1}, & if\;S=1\\ I_{0}, & if\;S=0 \end{array}\right..$$

(b)—The (2:1) TMUX in [38] has $C_{in}$ as a selector with values 0 or 1 ($V_{dd}/2$). A special (2:1) TMUX is presented in Figure 3, as shown in Equation (4).

(4) $$Z=\left\{\begin{array}{cc} I_{0}, & if\;C_{in}=0\\ I_{1}, & if\;C_{in}=1 \end{array}\right.$$

Compared to the standard (2:1) Binary MUX, which has two inputs (0 or Vdd), this special (2:1) Ternary MUX has two inputs (0 or Vdd/2). Moreover, the second difference is that the Cn is the output of the NTI of the selector $C_{in}$ instead of $\bar{C}$ in (2:1) Binary MUX.

## 4. Design Methodology

A 1-trit TFA sums three ternary inputs (*A*, *B*, and $C_{in}$ (Carry In)) and generates two outputs: the Sum and the Carry Out ($C_{out}$), as shown in Table 5. $C_{in}$ has only two values: 0 (0 V) and 1 ($V_{dd}/2$).

The general equations for the Sum and the Carry Out ($C_{out}$) are described in Equation (5):

(5) $$\begin{array}{c} Sum=\left(A+B+C_{in}\right)\;mod\left(3\right),\\ C_{out}=\left\lfloor \left(A+B+C_{in}\right)/3\right\rfloor . \end{array}$$

There are many methodologies to design TFAs. This paper uses unary operators with cascading TMUXs. Therefore, we derive Equations (6) and (7) from Table 5.

(6) $$Sum=\left\{\begin{array}{cc} A\cdot B_{0}+A^{1}\cdot B_{1}+A^{2}\cdot B_{2} & if\;C_{in}=0\\ A^{1}\cdot B_{0}+A^{2}\cdot B_{1}+A\cdot B_{2} & if\;C_{in}=1 \end{array}\right.,$$

(7) $$\begin{array}{c} C_{out}=\left\{\begin{array}{cc} 0\cdot B_{0}+\left(1\cdot \bar{A_{p}}\right).B_{1}+\left(1\cdot \bar{A_{n}}\right)\cdot B_{2} & if\;C_{in}=0\\ \left(1\cdot \bar{A_{p}}\right)\cdot B_{0}+\left(1\cdot \bar{A_{n}}\right)\cdot B_{1}+1.B_{2} & if\;C_{in}=1 \end{array}\right., \end{array}$$

where

(8) $$B_{i}=\left\{\begin{array}{cc} 2 & if\;B=i\\ 0 & if\;B\neq i \end{array}\right..$$

So, the input *B* will be the selector for (3:1) TMUXs and the input $C_{in}$ will be the selector for (2:1) TMUXs.

**Figure 4:**
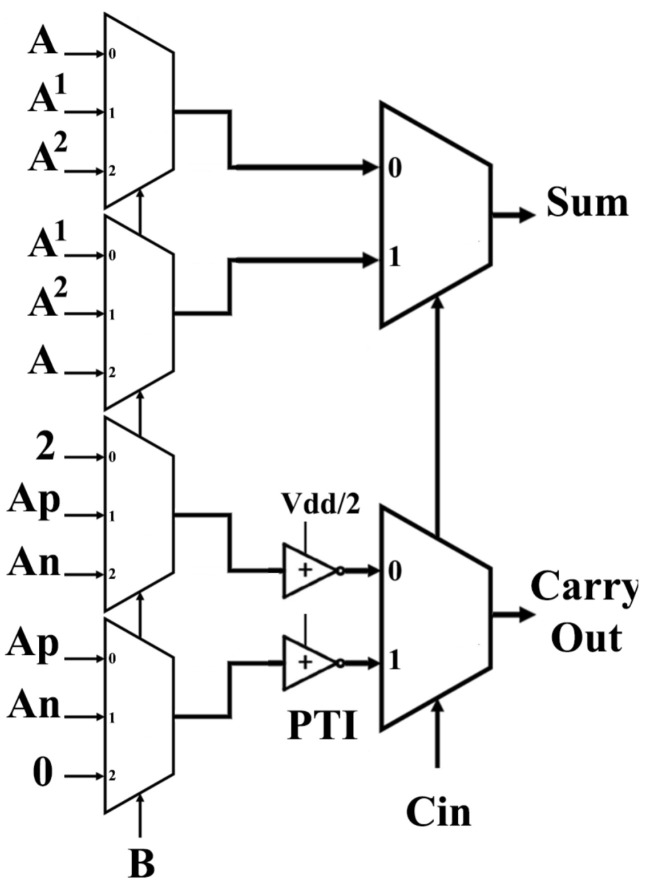
Proposed TFA with 68 CNTFETs (TMUX Model).

**Figure 5:**
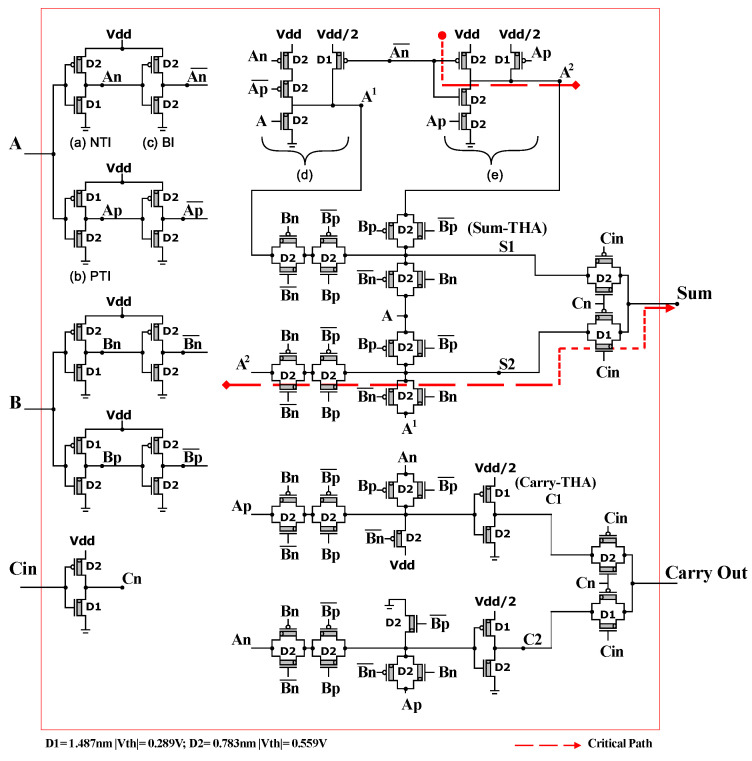
Proposed TFA with 68 CNTFETs (Transistor level). Unary operator sub-circuits are (**a**) NTI, (**b**) PTI, (**c**) binary inverter, (**d**) $A^{1}$, and (**e**) $A^{2}$.

Figure 4 and Figure 5 show the proposed 1-trit TFA circuit using unary operators and TMUXs.

The dotted red line (the critical path) represents the maximum propagation delay from the input “A” to the final output “Sum”.

### Operations of the Proposed TFA

The suggested design can be explained in five steps.

Step1: *A*, *B*, and $C_{in}$ are the inputs of the unary operators (NTI, PTI), resulting in An, Ap, Bn, Bp, and Cn as outputs.
Step2: An, Ap, Bn, and Bp are the inputs of the binary inverter, resulting in $\bar{An}$, $\bar{Ap}$, $\bar{Bn}$, and $\bar{Bp}$ as outputs.
Step3: *A*, An, Ap, $\bar{An}$, and $\bar{Ap}$ are the inputs of the two subcircuits Figure 5d,e, resulting in $A^{1}$ and $A^{2}$ as outputs.
Step4: *A*, $A^{1}$, and $A^{2}$ are the inputs of the two (3:1) TMUXs. Then, enter the (2:1) TMUXs to produce the SUM.
Step5: An, Ap, Vdd, and Ground are the inputs of the two (3:1) TMUXs. Then, enter two PTIs with (Vdd/2), resulting in $1\cdot \bar{A_{n}}$ and $1\cdot \bar{A_{p}}$ that are the inputs of the (2:1) TMUXs to produce the Carry Out.

## 5. Results and Comparison

The proposed TFA and the designated 17 circuits are simulated and compared using the HSPICE simulator.

Where these 17 circuits are divided as follows: 15 circuits with 32-nm-channel CNTFET-based TFAs in [30,31,32,33,34,35,36,37,38,39,40,41,42], one 2-bit binary FA FinFET-based design, and one 2-bit binary FA CMOS-based design in [29].

Note that we chose a 2-bit binary FA because the total number of combinations of a 1-trit TFA is 18 (see Table 5), and the number of inputs of a 2-bit binary FA is 4 (2 bits per input); then, the total number of combinations is 16 ($2^{4}$).

**Figure 6:**
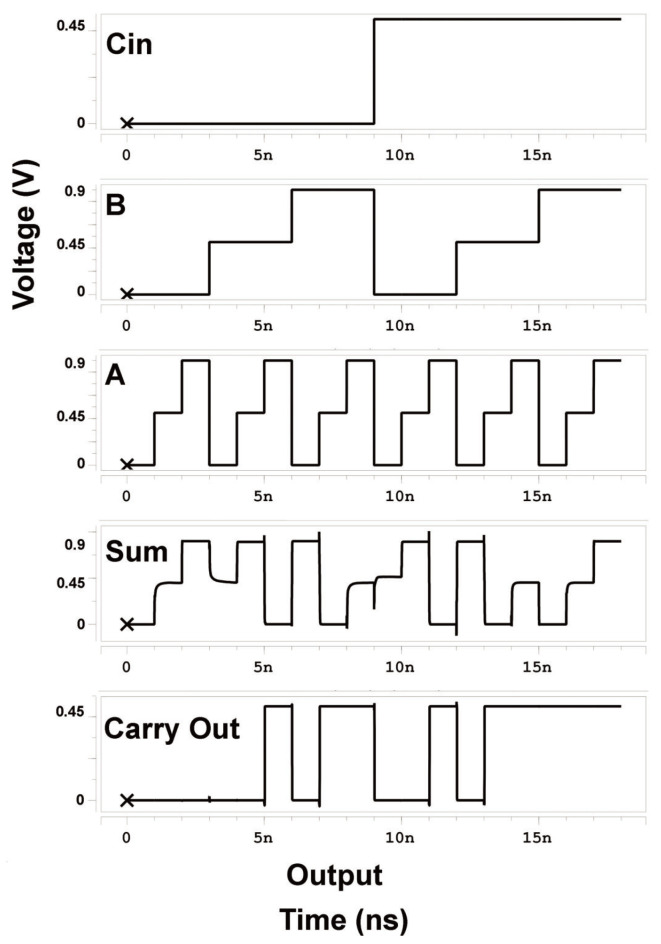
Waveform of the proposed TFA.

We unified all the simulation parameters in HSPICE for Table 6 and Figure 6 to $V_{dd}$ = 0.9 V, temperature = 27 C, frequency = 1 GHz, and fall/rise time = 20 ps for all input signals.

Figure 6 shows the HSPICE output waveform of the proposed TFA to verify the truth Table 5: Three ternary inputs (*A*, *B*, and $C_{in}$ (Carry In)) and two outputs: the Sum and the Carry Out ($C_{out}$).

Table 6 shows the comparison of all the studied circuits concerning the transistor count, average power, maximum delay, and maximum power delay product (PDP). The values in bold represent the best values.

**Figure 7:**
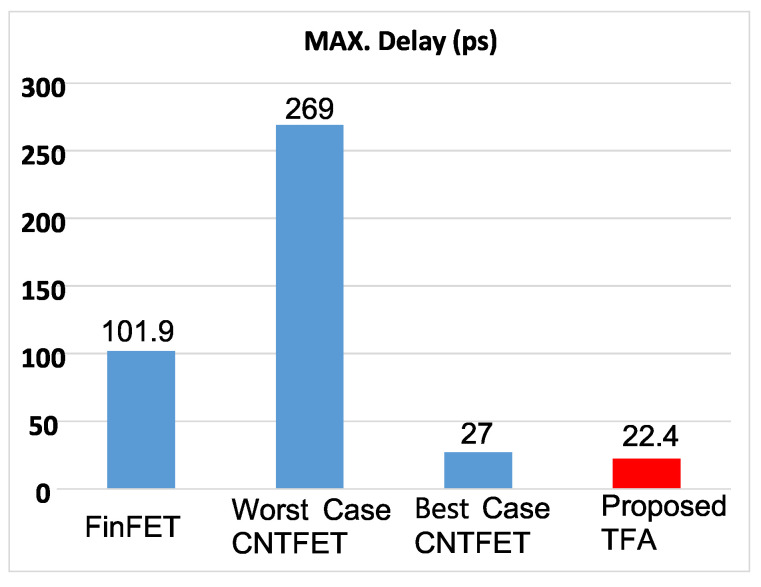
Bar chart comparison regarding the max. delay: the ternary best case CNTFET [38], the ternary worst case CNTFET [34], and the binary FinFET [29].

Figure 7 shows the bar chart comparison of the proposed TFA regarding the maximum delay with the ternary best case CNTFET [38], the ternary worst case CNTFET [34], and the binary FinFET [29].

The HSPICE results demonstrate that the proposed TFA is even better than the other best designs studied and implemented regarding the maximum propagation delays and PDP, which will affect the overall network performance by speeding up the CPU and therefore mitigating high CPU utilization. This proposed ternary adder succeeded in reducing the propagation delays by over 99% compared to the 2-bit binary FA CMOS-based design, over 78% compared to the 2-bit binary FA FinFET-based design, over 91% compared to the worst-case TFA, and over 49% compared to the best-case TFAs, which will enhance the CPU’s capabilities and has a significant positive impact on SDN. By enhancing the CPU, the execution of control plane tasks is accelerated, resulting in the faster and more efficient performance of the SDN controller software. This enables swift communication with network devices, quicker decision-making based on network state information, and the ability to handle complex network control applications. By addressing these limitations, SDN can achieve improved CPU performance and maximize the benefits of programmability, which introduces additional CPU overhead. Overall, an improved CPU empowers SDN with enhanced control, programmability, and agility in managing networks.

## 6. Conclusions

The CPU’s performance in an SDN controller is foundational to the network’s capability to function efficiently and adapt to new demands. By accelerating the speed of the CPU, the controller will be able to manage more network devices and handle larger volumes of traffic.

For the first time, this paper uses a ternary system within the CPU of the SDN controller to enhance the network management functionality. This paper proposed a new design of a 32 nm CNTFET-based TFA. The design process presented various techniques for transistor arrangement, two power supplies (Vdd, Vdd/2), and transistor count reduction, and it achieved the final target.

The HSPICE simulation results of the proposed circuit clearly show a better performance with lower propagation delays and energy consumption.

Finally, implementing a ternary system in the CPU of the SDN controller holds good promise for further advancement in network management in SDN architectures.

## Figures and Tables

**Table 1:** Aspects affecting programmability and CPU utilization.

Aspect	Programmability	CPU Utilization
Control logic flexibility	High—Easily modifiable	Moderate—Depends on complexity of tasks
Policy Update Frequency	High—Dynamic updates possible	High—Frequent updates increase CPU load
Algorithm Complexity	High—Complex algorithms can be implemented	High—Intensive computations require powerful CPU
Flow Management	Flexible—Can adjust flows dynamically	Moderate to High—Managing flow tables can be CPU-intensive
Network Analytics	Extensive—Detailed monitoring and reporting	High—Continuous data collection and analysis
Device Interfacing	Flexible—Can communicate with diverse devices	Moderate—Interfacing requires periodic CPU cycles

**Table 2:** Literature Review Summary.

Techniques	Ref.	Year	Details	CNTFET # TFA	Limitation
Binary Design	[29]	2023	- 2-bit Binary Full Adder	250	- High Propagation Delays
			- CMOS		- Medium transistor count
			- FinFET		- High PDP
The below references are using Ternary systems and CNTFETs					
Conventional Design	[30]	2011	- TDecoders (16 CNTFETs)	412	
			- Binary gates		
			- Ternary encoder		- High transistor count
	[31]	2021	- TDecoders (10 CNTFETs)	337	- High PDP
			- Binary gates		
			- 14 RRAMs		
Algorithms	[32]	2017	- Two custom Algorithms	105	
			- Cascading TMUXs		
	[33]	2018	- TBDD Algorithm	98	- High Propagation Delays
	[34]	2020	- Modified Quine-McCluskey Algorithm	106	- High PDP
TMUXs & Unary Operators	[35]	2017	- Two voltage supplies	74	- Cascading Transmission Gates
			- TMUXs (12 CNTFETs)		- High PDP & Propagation Delays
	[36]	2018	- Two voltage supplies	89	- High transistor count
			- TMUXs (22 CNTFETs)		
	[37]	2021	- TMUXs (15 CNTFETs)	72	
	[38]	2023	- 2 Designs	59	- Low transistor count
			- TMUXs (15 CNTFETs)	55	- Low PDP
			- Two voltage supplies		
Mixed Designs	[39]	2019	- Ternary encoders	142	
			- TMUXs (18 CNTFETs)		- High PDP & transistor count
			- Unary Operators based on Binary NAND		
	[40]	2020	- STI inverter	49	
			- Capacitive network	37	- Very High Propagation Delays
			- 2 Designs		- Very high PDP
	[41]	2021	- TMUXs (12 CNTFETs)	74	
			- PTL		
	[42]	2021	- TDecoders	54	- Medium PDP
			- Unary Operators		- Medium Propagation Delays
			- PTL		
			- Transmission Gates		

**Table 3:** Operations of the CNTFET.

Type	Diameter	Threshold Voltage	Voltage Gate		
			0 V	0.45 V	0.9 V
P-CNTFET	D1	−0.289 V	ON	ON	OFF
	D2	−0.559 V	ON	OFF	OFF
N-CNTFET	D1	0.289 V	OFF	ON	ON
	D2	0.559 V	OFF	OFF	ON

D1 = 1.487 nm, D2 = 0.783 nm.

**Table 4:** Selected Unary Operator Truths Table.

Ternary	NTI	PTI	Cycle Operators		Decisive		
Input A	$A_{n}$	$A_{p}$	$A^{1}$	$A^{2}$	Literal $A_{1}$	$1\cdot \bar{A_{p}}$	$1\cdot \bar{A_{n}}$
0	2	2	1	2	0	0	0
1	0	2	2	0	2	0	1
2	0	0	0	1	0	1	1

$A_{n}$: NTI (negative ternary inverter); $A_{p}$: PTI (positive ternary inverter); $A^{1}=\left(A+1\right)$ mod (3) is called the single shift operator or the successor; $A^{2}=\left(A+2\right)$ mod (3) is called the dual shift operator or the predecessor; $A_{1}$ is the decisive literal.

**Table 5:** TFA Truth Table.

$C_{\mathrm{in}}$	B	A	Sum	Carry Out
0	0	$\begin{array}{c} 0\\ 1\\ 2 \end{array}$	$\left.\begin{array}{c} 0\\ 1\\ 2 \end{array}\right\}A$	$\left.\begin{array}{c} 0\\ 0\\ 0 \end{array}\right\}0$
	1	$\begin{array}{c} 0\\ 1\\ 2 \end{array}$	$\left.\begin{array}{c} 1\\ 2\\ 0 \end{array}\right\}A^{1}$	$\left.\begin{array}{c} 0\\ 0\\ 1 \end{array}\right\}1\cdot \bar{A_{p}}$
	2	$\begin{array}{c} 0\\ 1\\ 2 \end{array}$	$\left.\begin{array}{c} 2\\ 0\\ 1 \end{array}\right\}A^{2}$	$\left.\begin{array}{c} 0\\ 1\\ 1 \end{array}\right\}1\cdot \bar{A_{n}}$
1	**0**	$\begin{array}{c} 0\\ 1\\ 2 \end{array}$	$\left.\begin{array}{c} 1\\ 2\\ 0 \end{array}\right\}A^{1}$	$\left.\begin{array}{c} 0\\ 0\\ 1 \end{array}\right\}1\cdot \bar{A_{p}}$
	1	$\begin{array}{c} 0\\ 1\\ 2 \end{array}$	$\left.\begin{array}{c} 2\\ 0\\ 1 \end{array}\right\}A^{2}$	$\left.\begin{array}{c} 0\\ 1\\ 1 \end{array}\right\}1\cdot \bar{A_{n}}$
	2	$\begin{array}{c} 0\\ 1\\ 2 \end{array}$	$\left.\begin{array}{c} 0\\ 1\\ 2 \end{array}\right\}A$	$\left.\begin{array}{c} 1\\ 1\\ 1 \end{array}\right\}$ **1**

**Table 6:** TFA Comparison.

Ref./Year	Transistor	Power	Max.	Max. PDP
	Count	(μW)	Delay (ps)	(×$10^{-18}$ J)
2-bit Binary Full Adder Using CMOS and FinFET				
In [29] 2023 *^a^*	250 CMOS	313.3	19790	6200 (×$10^{6}$)
In [29] 2023 *^b^*	250 FinFET	39.7	101.9	4.04 (×$10^{6}$)
1-trit Ternary Full Adder Using CNTFET				
In [30] 2011	412	1.36	88	120
In [31] 2021	337	1.96	78	153
In [33] 2018	98	0.16	192	31
In [34] 2020 *^c^*	106	**0.13**	269	35
In [32] 2017	105	1.13	68	77
In [35] 2017	74	0.82	146	120
In [36] 2018	89	0.44	48	21
In [37] 2021	72	0.28	51	14.3
In [39] 2019	142	4.62	94	434
In [40] 2020	49	1.23	192	236
In [40] Design 2	**37**	0.81	262	212
In [41] 2021	74	**0.13**	98	12.75
In [42] 2021	54	0.43	47	20
In [38] 2023 *^d^*	59	0.46	27	12.42
In [38] Design 2	55	0.22	34	7.48
**Proposed TFA**	68	0.28	**22.4**	**6.27**
Improvement				
w.r.t [29] ^*a*^ CMOS	−72.80%	−99.91%	−99.88%	−100%
w.r.t [29] *^b^* FinFET	−72.80%	−99.29%	−78.02%	−99.99%
w.r.t [34] *^c^*	−35.85%	+115.38%	−91.67%	−82.08%
w.r.t [38] *^d^* Design 1	+15.25%	−39.13%	−17.04%	−49.52%

*^a^* Compared to the binary CMOS circuit; *^b^* Compared to the binary FinFET circuit; *^c^* Compared to the highest propagation delay among all TFAs; *^d^* Compared to the lowest propagation delay among all TFAs.

## Data Availability

The original contributions presented in the study are included in the article, further inquiries can be directed to the corresponding author.

## Abbreviations

The following abbreviations are used in this manuscript:

CMOS	Complementary Metal-Oxide Semiconductor
CNFET	Carbon Nanotube Field-Effect Transistor
FinFet	Field-Effect Transistor
MVL	Multiple-Valued Logic
NTI	Negative Ternary Inverter
PDP	Power Delay Product
PTI	Positive Ternary Inverter
PTL	Pass Transistor Logic
RRAM	Resistive Random Access Memory
SDR	Software-Defined Radio
STI	Standard Ternary Inverter
STP	Spanning Tree Protocol
TBDD	Ternary-Transformed Binary Decision Diagram
TDecoder	Ternary Decoder
TFA	Ternary Full Adder
TMUX	Ternary Multiplexer
